# In Vivo Iterative Adjuvant Screening Identifies an Intranasal Vaccine Formulation for Elicitation of Protective Mucosal Immune Responses Against SARS-CoV-2

**DOI:** 10.3390/pharmaceutics17111422

**Published:** 2025-11-03

**Authors:** Yang Jiao, Sara H. Mahmoud, Chengjin Ye, Yuan Luo, Wei-Chiao Huang, Qinzhe Li, Shiqi Zhou, Yiting Song, Moriya Tsuji, Luis Martinez-Sobrido, Jonathan F. Lovell

**Affiliations:** 1 Department of Biomedical Engineering, State University of New York at Buffalo, Buffalo, NY 14260, USA; 2 Host-Pathogen Interactions (HPI) and Disease Intervention and Prevention (DIP) Programs, Texas Biomedical Research Institute, San Antonio, TX 78227, USA; sarahussein9@yahoo.com (S.H.M.);; 3 Center of Scientific Excellence for Influenza Viruses, National Research Centre, Giza 12622, Egypt; 4 Aaron Diamond AIDS Research Center, Division of Infectious Diseases, Department of Medicine, Columbia University Irving Medical Center, New York, NY 10032, USA

**Keywords:** vaccines, liposomes, SARS-CoV-2, intranasal, adjuvants, RBD

## Abstract

**Background:** Intranasal (I.N.) vaccination holds promise to elicit mucosal immunity that counters respiratory pathogens at the site of infection. For subunit protein vaccines, immunostimulatory adjuvants are typically required. **Methods:** We screened a panel of 22 lipid-phase adjuvants to identify which ones elicited antigen-specific IgA with I.N. immunization of liposome-displayed SARS-CoV-2 receptor-binding domain (RBD). **Results:** Initial screening showed the TLR-4 agonist Kdo2-Lipid A (KLA) effectively elicited RBD-specific IgA. A second round of screening identified further inclusion of the invariant NKT cell ligands α-Galactosylceramide (α-GalCer) and its synthetic analog 7DW8-5 as complementary adjuvants for I.N. immunization, resulting in orders-of-magnitude-greater mucosal IgA response relative to intramuscular (I.M.) immunization. The inclusion of cationic lipids conferred capacity for mucosal adhesion and maintained immune responses. In K18 hACE2 transgenic mice, vaccination significantly reduced viral replication and prevented mortality from SARS-CoV-2 challenge. **Conclusions:** These results point towards the potential for the use of KLA and α-GalCer for I.N. subunit vaccines.

## 1. Introduction

Severe acute respiratory syndrome coronavirus 2 (SARS-CoV-2) caused a global health crisis, prompting extensive efforts to develop effective vaccines [1]. While intramuscular (I.M.) vaccines have demonstrated strong systemic immune responses, their ability to induce mucosal immunity in the respiratory tract remains limited [2]. Given that SARS-CoV-2 primarily enters the body through the mucosal surfaces of the upper respiratory tract, intranasal (I.N.) vaccination offers a promising alternative by inducing local immune responses, including secretory IgA production, which can provide superior protection against viral transmission and infection [3,4].

I.N. vaccines have been explored for their ability to generate both mucosal and systemic immunity, offering advantages such as ease of administration and enhanced immune response at the site of viral entry [5]. Various strategies have been employed to improve the efficacy of I.N. vaccines, including the use of adjuvants to enhance immunogenicity [6,7]. For example, I.N. vaccines for SARS-CoV-2 have been developed with mRNA [8], polymers [9], and exosomes [10]. Some viral vector I.N. vaccines (e.g., iNCOVACC and dNS1-RBD) have been used in human trials [11,12]. dNS1-RBD is a nasal spray vaccine developed using a modified H1N1 influenza virus that lacks the NS1 gene that carries the receptor-binding domain (RBD) of the SARS-CoV-2 spike (S) protein as the antigen. It has shown effectiveness in inducing both systemic and mucosal immune responses and has been authorized for emergency use in China for adult booster shots [13]. iNCOVACC (BBV154) is an I.N. COVID-19 vaccine developed by Bharat Biotech that uses a chimpanzee adenovirus vector (ChAd36) to deliver the stabilized S protein of SARS-CoV-2. However, achieving robust mucosal immunity often requires adjuvants. Studies have shown that I.N. subunit vaccines co-administered with Toll-like receptor (TLR) agonists can enhance mucosal antibody responses [14]. Similarly, a vaccine formulated with Bacterial Enzymatic Combinatorial Chemistry (BECC)-derived adjuvant BECC470 and delivered I.N. was shown to elicit strong systemic and localized immune responses in K18 hACE2 transgenic mice, providing protection against infection with SARS-CoV-2 [15]. In combating post-acute sequelae of COVID-19, a nasal mucoadhesive nanoformulation, EC16m, could terminate the persistent infection in the olfactory epithelium and restore the olfactory function [16].

Liposome-based vaccines have emerged as a potential platform for antigen presentation [17]. These lipid-based carriers can encapsulate antigens, enhancing their delivery to antigen-presenting cells (APC) and subsequently boosting the immune response [18,19]. In our prior studies, we developed cobalt porphyrin–phospholipid (CoPoP) liposomes, which can facilitate the stable display of recombinant antigens, enabling efficient uptake by APC and the induction of strong humoral and cellular immune responses [20,21]. CoPoP liposomes displaying the RBD of the SARS-CoV-2 S glycoprotein have been shown to elicit robust neutralizing antibody responses and provide protective immunity in mouse models [21,22,23] and human trials [24,25].

In this study, we adopted a previously proposed in vivo adjuvant screening approach that maximized CD8^+^ T-cell elicitation with short peptides and intramuscular injection [26]. In this case, we aimed to optimize the immunogenicity of an I.N. CoPoP liposome-displayed RBD nanoparticle vaccine by screening 22 different adjuvant formulations. The combination of Kdo2-Lipid A (KLA) and α-Galactosylceramide (α-GalCer) emerged as a top candidate from the screening. KLA, a synthetic analog of lipid A, serves as a potent Toll-like receptor 4 (TLR4) agonist, enhancing vaccine immunogenicity by stimulating APC and inducing pro-inflammatory cytokine production [27,28]. α-GalCer is a glycolipid that has been shown to activate invariant natural killer T (iNKT) cells through CD1d-mediated antigen presentation, leading to rapid cytokine release and modulation of immune responses [29,30,31].

Cationic biomaterials, such as chitosan, have been widely explored as mucoadhesive adjuvants due to their positive charge that interacts with negatively charged mucosal surfaces, ability to prolong antigen retention at mucosal surfaces, and favorable biocompatibility and safety profile [32]. This provides a conceptual basis for assessing positively charged lipids in the formulation. The electrical charge of lipids significantly influences the characteristics and applications of liposomes [33]. It has been reported that cationic liposomes have mucoadhesive properties and can enhance the efficacy of liposome-based vaccines [34,35]. Therefore, we investigated the impact of different charged lipids on mucoadhesion and antigen retention in the respiratory tract. Our findings provide insights into the development of an effective I.N. vaccine strategy for SARS-CoV-2, with potential applications for other respiratory pathogens.

## 2. Materials and Methods

Materials: The adjuvants used in screening and their vendors are shown in Table S1. Recombinant SARS-CoV-2 RBD from the Wuhan strain, expressed in human embryonic kidney 293 cells (HEK293), was obtained from Raybiotech (Cat# 230-30162, Peachtree Corners, GA, USA). Proteins were dialyzed into phosphate-buffered saline (PBS) to remove any trace imidazole. 1,2-dioleoyl-sn-glycero-3-phosphocholine (DOPC) was obtained from Corden Pharma (Cat# LP-R4-070, Basel, Switzerland). 1,2-dioleoyl-3-trimethylammonium-propane (DOTAP), 1,2-dioleoyl-sn-glycero-3-phosphoglycerol (DOPG), and Phyto Cholesterol (Chol) were obtained from Wilshire Technologies (Cat# 57-88-5, Princeton, NJ, USA). CoPoP and PoP were produced as previously described [23]. Mucin from bovine submaxillary glands was obtained from Sigma-Aldrich (Cat# M3895, St. Louis, MO, USA).

Liposome preparation: Liposomes were prepared by ethanol injection and nitrogen-pressurized lipid extrusion. Ethanol and PBS were preheated at 55 °C. Lipids (DOPC, cholesterol, and CoPoP) were dissolved in preheated ethanol for 10 min, followed by adding preheated PBS for another 10 min at 55 °C. Liposomes were then passed through 200, 100, and 80 nm stacked polycarbonate filters in a lipid extruder (Northern Lipids) with nitrogen pressure. After extrusion, liposomes were dialyzed against PBS to remove ethanol. Final liposome concentration was adjusted to 320 μg/mL CoPoP, passed through a 0.2 μm sterile filter, and stored at 4 °C. The CA liposome formulation has a mass ratio of [DOPC:Chol:CoPoP] = [20:5:1]. Other formulations, including DOTAP/CoPoP liposomes, have a lipid mass ratio of [DOPC:DOTAP:Chol:CoPoP] = [10:10:5:1], and DOPG/CoPoP liposomes have a lipid mass ratio of [DOPC:DOPG:Chol:CoPoP] = [10:10:5:1].

Particle size: Liposomes were incubated with SARS-CoV-2 RBD for 3 h at room temperature. After 500-fold dilution into PBS, samples were measured by dynamic light scattering (DLS) with a Nanobrook 90Plus PALS instrument (Brookhaven Instruments Corporation, Holtsville, NY, USA).

Fluorescence protein quenching assay: The procedures of labeling SARS-CoV-2 RBD with DY-490-NHS-Ester (Dyomics GmbH, Jena, Germany, Prod#: 490-01) used in this study were identical to those described in our previous work [23]. Fluorescence-labeled SARS-CoV-2 RBD was mixed with liposomes at a final concentration of 1 µg/mL in 96-well plates and incubated at room temperature for 3 h. Then, the fluorescence measurements were performed using a Tecan Safire microplate reader instrument (Tecan Group Ltd., Männedorf, Switzerland) with an excitation wavelength of 490 nm and an emission wavelength of 515 nm. The binding percentage was determined using the following equation:

*Binding%* = (1 − *Fluorescence*_post-incubation_)/*Fluorescence*_pre-incubation_ × 100%

Murine immunization: All procedures were carried out under protocols approved by the University at Buffalo IACUC. Outbred female Institute of Cancer Research (ICR) mice (CD-1; Envigo) were equally immunized I.N or I.M. on day 0 and day 14. The vaccines were prepared in a 50 µL volume and delivered via I.M. injection in the right hind leg. For I.N. administration, isoflurane-anesthetized mice were held upright, and 25 µL of vaccine was applied to each nare. In the first adjuvant screening, each dose contained 2 µg of SARS-CoV-2 RBD, 8 µg of CoPoP, 160 µg of DOPC, 40 µg of cholesterol, and 2 µg of candidate adjuvant. In the second round of screening, an extra 2 µg of Kdo_2_-Lipid A was added to this formulation. The formulation of charged lipid screening is shown in Table S1. SARS-CoV-2 RBD was mixed with liposome and incubated at room temperature for 3 h before immunization, then stored at 4 °C until use. Mice were sacrificed on day 28, and the sera and lungs were collected. Excised lungs were placed into RINO^®^ Sterile 1.5 mL Microcentrifuge Tube (Next Advance, SKU: TUBE1R5-S) containing three 2.3 mm stainless steel beads (Next Advance, SKU: SSB23) and 1.5 g of 0.9–2.0 mm blend stainless steel beads (Next Advance, SKU: SSB14B). Then, 700 µL of PBS was added, and the tissues were homogenized using Bullet Blender Storm 24 (Next Advance) for 10 min. The homogenates were centrifuged at 2000× *g* for 10 min at 4 °C, and the supernatant was collected for ELISA, neutralization antibody test, and pseudotyped virus (PSV) assay.

Enzyme-linked immunosorbent assay (ELISA): A total of 1 µg/mL of SARS-CoV-2 RBD in coating buffer (28.5 mM Na_2_CO_3_; 71.4 mM NaHCO_3_, pH 9.6) was coated on 96-well plates at 37 °C for 1 h. Tween-20 (0.05%) in PBS (PBST) was used to wash the 96-well plates 2 times. Then, plates were blocked with PBST containing 2% BSA at 37 °C for 1 h. The lung homogenates were diluted 5 to 625 times, and sera were diluted 10^2^ to 10^7^ times in 1% BSA in PBST, respectively, in 96-well plates. After incubating at 37 °C for 1 h, plates were washed with PBST 4 times. Goat anti-mouse IgA-HRP (SouthernBiotech, Birmingham, AL, USA, Cat#: 1040-05) was diluted 1500-fold in 1% BSA in PBST and incubated at 37 °C for 30 min. When testing the anti-RBD IgG titer, anti-mouse IgG- and HRP-linked antibodies (Cell Signaling Technology, Danvers, MA, USA, Cat# 7076S) were used instead of goat anti-mouse IgA-HRP, followed by washing the plates with PBST 6 times. A total of 100 µL of tetramethylbenzidine solution (Surmodics, Eden Prairie, MN, USA, Cat# TMBC-0100-01) was added to each well. After 3 min, 100 µL of 0.1M hydrochloric acid was added to stop the reaction. Antibody titers were determined as the reciprocal serum dilution at which the absorbance at 450 nm exceeded background by more than 0.5 absorbance units.

RBD-hACE2 Inhibition Assay: cPass™ SARS-CoV-2 Neutralization Antibody Detection Kit (GenScript, Piscataway, NJ, USA, Cat#: L00847) was used following the manufacturer’s instructions.

Pseudotyped virus (PSV) assays: HEK293T-hACE2 cells were seeded into 96-well plates at a density of 2 × 10^5^ cells/well overnight. Immunized mouse sera with serial dilutions were incubated with PsV at RT for 30 min, then 50 µL of Psv with sera at different serum dilutions were added to each well after removing 50 µL of culture medium, and the cells were cultured for 48 h. The medium was removed from each well, and the cells were washed with 200 µL PBS, followed by adding 30 µL of lysis buffer (Promega E1500, Madison, WI, USA) for 10 min. The lysate was transferred into a white plate, and 100 µL of substrate was added. CentroPRO (Cat. # LB962, Berthold Technologies, Bad Wildbad, Germany) was used to measure luciferase activity.

Zeta potential: Samples were diluted in DI water, then ζ-potential and particle size were measured by Zetasizer-Malvern Panalytical Advance Ultra (Malvern Panalytical Ltd., Malvern, Worcestershire, UK).

Mucoadhesion: In total, 0.1 mL of CA/DOPC, CA/DOTAP, or CA/DOPG samples was mixed with 0.3 mL of different concentrations of mucin solution (0–1 mg/mL), respectively, followed by incubation at 37 °C for 20 min. Then, samples were centrifuged at 8000× *g* for 5 min. A total of 20 µL of the supernatant was taken and diluted 50-fold in ethanol, and the absorbance at the 645 nm Q-band of CoPoP was measured with a Lambda 365 UV-VIS spectrophotometer instrument (PerkinElmer Inc., Shelton, CT, USA). The percentage of nanoparticles fixed by mucin was calculated by the following equation:

*Nanoparticles fixed by mucin%* = (*A*_645nm of sample without mucin_ − *A*_645nm of sample with mucin_)/*A*_645nm of sample without mucin_ × 100%

Mice immunization and SARS-CoV-2 challenge: Animal studies were carried out according to protocols approved by Texas Biomedical Research Institute (TBRI). K18-hACE2 mice (strain # 034860; Jackson Laboratories, Bar Harbor, ME, USA) (*n* = 13), aged 6–8 weeks, received intranasal immunizations on days 0 and 14. The vaccine was prepared in a 50 µL volume and delivered via intranasal isoflurane-anesthetized mice were held upright, and 25 µL of vaccine was applied to each nare. Serum samples were collected on days 0, 14, and 28 days post-vaccination, then stored at −20 °C to assess serum antibodies against SARS-CoV-2 RBD protein. Neutralizing titers were determined against infectious SARS-CoV-2 in a plaque reduction neutralization assay.

To assess protection efficacy, two weeks after the second immunization, mice were intranasally inoculated with 10^5^ PFU of rSARS-CoV-2/USA WA1 Nluc isolate (expressing nLuc) in 50 μL of sterile PBS while under anesthesia. Body weight was recorded daily, with the initial weight set as 100%. In survival experiments, mice were monitored every 24 h. Animals showing lethargy, moribundity, or >20% loss of initial body weight were humanely euthanized and considered to have succumbed to infection for survival analysis purposes. For the necropsy groups, nasal turbinate and lungs were collected on days 2 and 4 after the challenge to assess the presence of the virus and to evaluate pathology in the lungs. Mock (PBS)-vaccinated mice challenged with rSARS-CoV-2, and unvaccinated and mock-challenged mice were included as internal controls.

For in vivo bioluminescence imaging, mice were anesthetized with ketamine, injected retroorbitally with 100 μL of Nano-Glo luciferase substrate (Promega), and immediately imaged. The bioluminescence data acquisition and analysis were performed using the Aura program (AMI spectrum). The scale used is included in each figure. Immediately after imaging, nasal turbinate and lungs were collected and homogenized in 1 mL of PBS. Supernatants were collected, and the presence of virus was determined by plaque assay. Nluc activity in the TCSs was determined under a multiplate reader (BioTek Instruments, Inc., Winooski, VT, USA).

SARS-CoV-2 virus titrations: Nasal turbinate and lungs from SARS-CoV-2-infected mice were homogenized in 1 mL of PBS for 20 s at 7000 rpm using a Precellys tissue homogenizer (Bertin Instruments, Daytona Beach, FL, USA). Confluent monolayers of Vero AT cells (24-plate format, 4 × 10^4^ cells/well) were infected with 10-fold serial dilutions of supernatants obtained from the nasal turbinate or lung homogenates from SARS-CoV-2-infected mice. Virus from serially diluted samples was adsorbed at 37 °C for 1 h. After viral adsorption, cells were overlaid with post-infection media containing 1% low-melting agar and were incubated at 37 °C for 72 h. After viral infection, plates were submerged in 10% NBF for 24 h for fixation/inactivation. Plaque visualization was performed using crystal violet staining. After fixation, the overlay was removed, and cells were washed twice with PBS. Crystal violet solution (0.5% crystal violet in 20% ethanol) was added to the wells and incubated for 30 min. The plates were gently washed with tap water to remove excess stain, and plaques were visualized as clear zones against the stained monolayer.

Plaque reduction microneutralization (PRMNT) assays: PRMNT assays were performed to identify the levels of SARS-CoV-2 NAbs as described previously [36] Briefly, serum samples were inactivated at 56 °C for 30 min prior to assay. For pre-treatment conditions, ~100–200 PFU/well of SARS-CoV-2 was mixed with 2-fold serial dilutions (starting dilution was 1:10 for serum samples collected at 14 d.p.p. and 14 d.p.b for collected sera) of mice’ sera and incubated at 37 °C for 1 h. Confluent monolayers of Vero AT cells (96-well plate format, 4 × 10^4^ cells/well, quadruplicates) were infected with the virus–serum mixture for 1 h at 37 °C. After viral absorption, infectious media were exchanged with post-infection media containing 2% FBS and 1% Avicel. Infected cells were fixed 14 h post-infection (hpi) with 10% neutral formalin for 24 h and immunostained with a monoclonal Ab (1C7) against the viral nucleocapsid (N) protein (1 μg/mL). Viral neutralization was evaluated and quantified using ELISPOT, and a sigmoidal dose–response, non-linear regression curve was generated using Prism 10 (GraphPad Prism 10, San Diego, CA, USA) to calculate median neutralization titer (NT_50_) in each of the serum samples.

## 3. Results

### 3.1. KLA/7DW8-5 and KLA/α-GalCer Formulation Vaccines Induced High Levels of Anti-RBD IgA Titer in Lung

**Figure 1:**
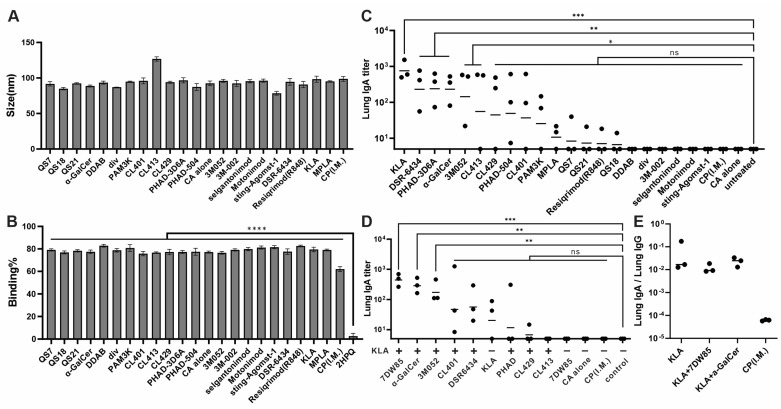
Adjuvant screening reveals those that elicit mucosal antibody responses in mice: (**A**) Diameter of particles with different adjuvants. (**B**) Fluorescence quenching of SARS-CoV-2 RBD reflecting binding to adjuvanted CoPoP or PoP liposomes (that are identical but lack cobalt). Statistical comparison based on one-way ANOVA with Dunnett’s multiple comparison. (**C**) Outbred ICR mice were immunized with I.N. on days 0 and 14, and serum and lung homogenate were collected on day 28 for analysis. Log10-transformed anti-RBD IgA titer was measured with lung homogenates by ELISA and analyzed based on one-way ANOVA with Dunnett’s multiple comparisons. (**D**) Similar immunization was performed but with KLA adjuvanted liposomes also containing additional adjuvants as indicated. (**E**) The ratio of Lung IgA titer to Lung IgG titer. *n* = 3 mice per group. * *p* < 0.05; ** *p* < 0.01; *** *p* < 0.001; **** *p* < 0.0001; ns: not statistical.

We mixed His-tagged SARS-CoV-2 RBD with aqueous CoPoP/CHOL/DOPC liposome (CoPoP alone; abbreviated herein as “CA”) and different candidate adjuvants to generate a liposome-displayed RBD nanoparticle. DLS showed that the SARS-CoV-2 RBD mixed with CA and different candidate adjuvants formed stable nanoparticles with a diameter of around 100 nm (Figure 1A). When fluorescently labeled RBD was incubated with CA and different adjuvants, about 80% of the fluorescence was quenched after 3 h (Figure 1B), indicating that nearly 80% of SARS-CoV-2 RBD rapidly bound to liposomes as indicated by quenching and formed nanoparticles spontaneously based on their interaction with CoPoP. In contrast, SARS-CoV-2 RBD does not bind to PoP liposomes that are identical to CoPoP but lack cobalt.

Next, outbred ICR mice (*n* = 3/group) were immunized I.N. with 2 µg SARS-CoV-2 RBD nanoparticle vaccines on day 0 and boosted on day 14. The 2 µg RBD dose vaccine contained 8 µg of CoPoP, 0.16 mg of DOPC, and 2 µg of different adjuvants in a 50 µL aqueous solution. We screened a total of 22 lipid-phase immunostimulatory adjuvants as listed in Table S1, selected based on their theoretical ability to integrate into lipid bilayers and their diverse mechanisms of action [26]. A group of mice was immunized I.M. with CoPoP/PHAD and SARS-CoV-2 RBD served as a control. The mice were sacrificed on day 28, and the sera and lungs were collected. The anti-RBD IgA titer in lung homogenates was then assessed by ELISA. Figure 1C shows that the CA/KLA vaccine induced the highest IgA titers against SARS-CoV-2 RBD in the lung homogenate, followed by DSR-6434, PHAD-3D6A, α-GalCer, 3M052, CL413, and CL429. The relationship between IgA and IgG titers are presented for each adjuvant in Figure S1A,B. Meanwhile, the I.M. administration did not induce detectable RBD-specific IgA titer in lung homogenates. CA/KLA vaccine also induced a high anti-RBD IgG titer both in serum and lung homogenates, while CA/α-GalCer had the highest IgG titer of all I.N. administration groups (Figure S1C). Therefore, we picked KLA as the base adjuvant of our vaccine and carried another round of in vivo adjuvant screening. Next, 2 µg of the top-performing adjuvants from the first screening were added to 2 µg of the CA/KLA vaccine for the second round of screening. A derivative of α-GalCer, 7DW8-5, was included in the screening since it has been studied for its immunostimulatory properties and potential as a vaccine adjuvant [37,38]. In Figure 1D, ELISA results from lung homogenates show that the combination of KLA and 7DW8-5 induced the highest anti-RBD IgA titers in the lungs. Notably, 7DW8-5 was not effective on its own, but when used synergistically with KLA, its efficacy was markedly improved. The combination of KLA and α-GalCer vaccine has the second-highest IgA titer. These two combinations also induced the highest RBD-specific IgG titer in the lungs of all I.N. immunized mice (Figure S1D). We chose these two adjuvant combinations for the following research. We also measured the anti-RBD IgG titer in serum, and the result shows a pattern consistent with the IgA titer (Figure S1E,F). The I.M immunization induced the highest anti-RBD IgG titer in mice by far, but without a lung IgA response. Figure 1E shows that I.N. vaccination with optimal formulations preferentially elicited mucosal IgA responses, as reflected by orders-of-magnitude higher lung RBD-specific IgA/IgG titer ratios relative to I.M. vaccination.

### 3.2. Volume Consideration for I.N. Vaccine Administration 

**Figure 2:**
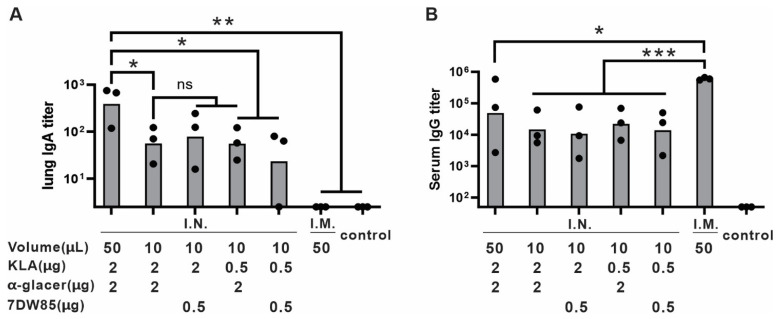
Adjuvant ratio study: Outbred ICR mice (*n* = 3/group) were immunized with vaccine formulations containing varying ratios of KLA and α-GalCer and total injection volume as indicated. On day 14 post-primary immunization, mice received a booster injection with the same formulation and volume. Serum and lung homogenate were collected on day 28 for ELISA analysis. Anti-RBD IgA titer measured with lung homogenate (**A**) and anti-RBD IgG titer (**B**) measured with serum and analyzed by ordinary one-way ANOVA with Dunnett’s multiple comparison. *n* = 3 mice per group; * *p* < 0.05; ** *p* < 0.01; *** *p* < 0.001; ns: not statistical. The control group refers to the untreated.

To further optimize the adjuvants formulation, different ratios and volumes of KLA and 7DW8-5 or KLA and α-GalCer formulation were tested to evaluate their impact on immune responses. Figure 2A shows that 2 µg of KLA resulted in higher lung IgA titers than 0.5 µg. The vaccine with 50 µL per dose induced higher lung IgA and serum IgG titers compared to the 10 µL formulation, despite having the same dosage otherwise (Figure 2A,B). This difference in volume may influence antigen dispersion, delivery and immune activation during I.N. immunization. There was no significant difference between different ratios of KLA to 7DW8-5 or α-GalCer in dosage. Based on these results, the optimized formulation contained a balanced combination of KLA and α-GalCer in 50 µL of aqueous solution for I.N. administration.

### 3.3. Cationic Lipid Formulations Provide Mucoadhesion and Maintain Immunogenicity

**Figure 3:**
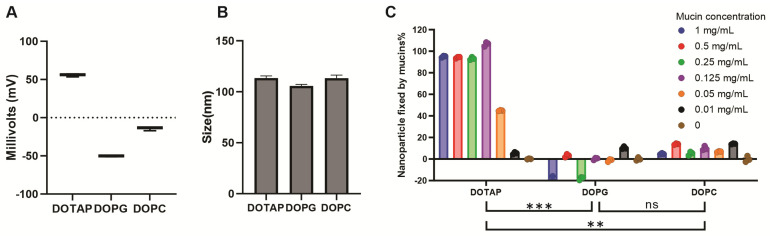
Cationic CoPoP liposome formulations bind to purified mucin: (**A**) ζ-potential and (**B**) the size of DOPG, DOPC, and DOTAP formulation liposomes. (**C**) Adhesion of formulations to different concentrations of mucins. Percentage of nanoparticles with varied surface charges fixed by mucin at decreasing concentrations (1, 0.5, 0.25, 0.125, 0.05, and 0.01 mg/mL). Statistical comparison based on a one-way ANOVA test followed by Tukey’s multiple comparison test at each concentration. ** *p* < 0.01; *** *p* < 0.001; ns: not statistically significant.

To evaluate the impact of lipid charge on mucoadhesion and nanoparticle stability, different formulations containing DOPC (neutral), DOTAP (positively charged), and DOPG (negatively charged) were tested. Figure 3A shows the ζ-potential and particle size of each formulation. The inclusion of DOTAP resulted in a more positive ζ-potential, while DOPG led to a more negative charge, confirming the expected electrostatic properties of these formulations. The nanoparticle size was also measured based on dynamic light scattering of three lipid formulations. The result indicates that their size remains approximately 100 nm (Figure 3B). Figure 3C demonstrates the adhesion of nanoparticles to mucin at varying concentrations. The formulations containing DOTAP (positively charged lipids) exhibited the highest mucin adsorption. These results suggest that positively charged lipids can enhance mucoadhesion while maintaining nanoparticle stability, potentially contributing to improved antigen retention in the respiratory tract and immune response following I.N. vaccination.

**Figure 4:**
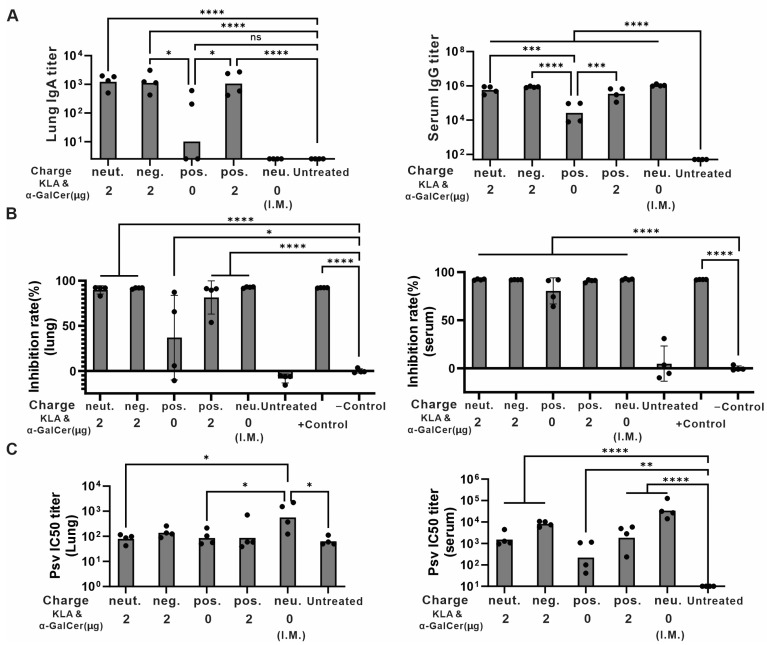
Murine study of different charged liposomal vaccines: (**A**) Anti-RBD lung homogenate IgA titer (left) and serum IgG titer (right). (**B**) The percent signal inhibition for the detection of neutralizing antibodies in lung homogenate (left) and serum (right) measured using the cPass SARS-CoV-2 Neutralizing Antibody Detection Kit. Inhibition rate% = (1 − OD value of Sample/OD value of Negative Control) × 100% (**C**) SARS-CoV-2 S pseudotyped virus (PSV) assay. Log10-transformed IgA titer (A left), IgG titer (A right), IC50 titer (**C**), and inhibition rate (**B**) were analyzed based on a one-way ANOVA test followed by Tukey’s multiple comparison test. Neut. refers to DOPC formulation; neg. refers to DOPG formulation; pos. refers to DOTAP formulation. *n* = 4 mice per group; * *p* < 0.05; ** *p* < 0.01; *** *p* < 0.001; **** *p* < 0.0001; ns: not statistical.

To assess the effect of lipids with different charges on their ability to induce immunogenicity in mice, ICR mice (*n* = 4/group) were immunized with various liposome compositions with varying charge, as detailed in Table S2. Four of these groups were immunized via I.N. administration, and a control was I.M.-immunized. Anti-RBD IgA and IgG titers in lung homogenates and serum were measured to evaluate immune responses (Figure 4A). With I.N. immunization, the groups that contained KLA/α-GalCer showed significantly higher IgA and IgG titers than other groups, regardless of liposome charge. The cPass neutralization assay showed that more than 90% of the interaction between RBD and ACE2 was inhibited by lung homogenates in G1, G2, and G5 (Figure 4B), which corresponds to the previous results showing functional antibody elicitation. Vaccines that included DOTAP lipids did not show higher immune responses compared to neutral ones. Thus, while a cationic charge was necessary for binding to mucin in vitro, further studies are needed to demonstrate under which conditions this could provide benefit for mucosal vaccination with improved IgA titers. When measured by post-immune sera, all groups had a high inhibition rate except for the group that lacked adjuvants. Additionally, the SARS-CoV-2 S pseudotyped virus (PSV) assay (Figure 4C) demonstrated potent neutralization activity in the sera from mice vaccinated with CoPoP liposomes administered by I.M. compared to the untreated group. Taken together, we selected the adjuvanted DOTAP formulation as the optimized vaccine for subsequent studies.

### 3.4. I.N. Vaccination Suppresses SARS-CoV-2 Replication and Prevents Disease Progression in K18-hACE2 Mice

**Figure 5:**
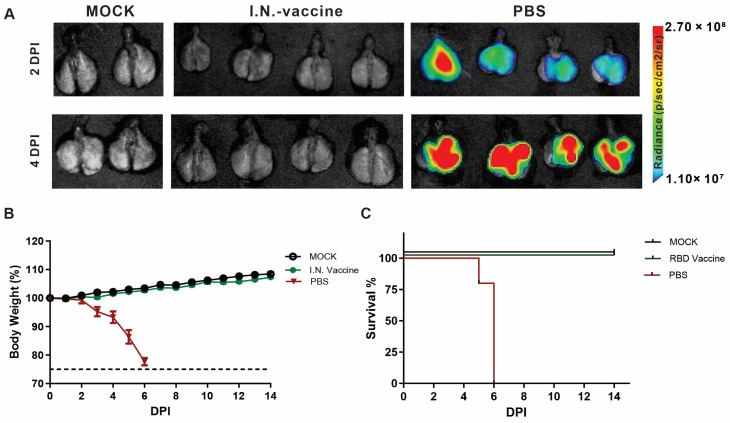
Replication and spread of rSARS-CoV-2 WA1-Nluc in vaccinated K18 hACE2 transgenic mice. Nluc activity in live mice (*n* = 4/group) (mock infected or infected (10^5^ PFU/mouse) with rSARS-CoV-2-WA1-Nluc) was determined at 2 and 4 dpi using the Ami HT IVIS. An in vivo Nluc expression representative image of the entire mouse at each time point is shown in Figure S3. (**A**) Ex vivo Nluc activity: Excised lungs from mock-infected or infected mice were monitored for Nluc activity at 2 and 4 dpi. Responses and protection in K18 hACE2 transgenic mice immunized with I.N. vaccine. Furthermore, 6–8-week-old K18 hACE2 transgenic mice (*n* = 5/group) were mock infected or I.N. inoculated with 10^5^ PFU/mouse of rSARS-CoV-2-WA1-Nluc. Body weight loss (**B**) and survival (**C**) after viral challenge were monitored for 14 days after viral infection.

To evaluate the protective efficacy of the optimized vaccine formulation, K18 hACE2 transgenic mice were immunized I.N. and challenged with 10^5^ PFU of rSARS-CoV-2-WA1-Nluc. The progression of rSARS-CoV-2-WA1-Nluc infection in vaccinated and unvaccinated K18 hACE2 transgenic mice was monitored using an Ami HT in vivo imaging system (IVIS). Ex vivo analysis of lungs revealed that unvaccinated mice exhibited robust Nluc activity as early as 2 days post-infection (dpi), which continued to increase at 4 dpi. In contrast, vaccinated mice showed negligible Nluc signal at all time points (Figure 5A), indicating that viral infection was effectively inhibited.

Clinical results showed that unvaccinated mice rapidly lost weight starting at 4 dpi after vaccination (Figure 5B), and all died by 6 dpi (Figure 5C). In contrast, vaccinated mice maintained stable weight and fully survived over the 14-day observation period. These results demonstrate that the vaccine is able to prevent severe disease and death.

**Figure 6:**
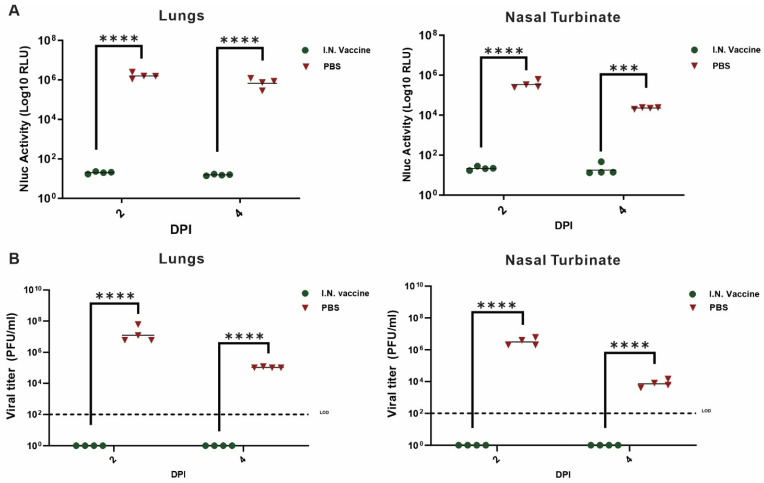
Nluc activity and viral titers in lung and nasal turbinate tissue homogenates from vaccinated K18 hACE2 transgenic mice: lungs (left) and nasal turbinate (right) of 6-8-week-old K18 hACE2 transgenic mice (*n* = 4/group) mock infected or infected I.N. with 10^5^ PFU/mouse with rSARS-CoV-2-WA1-Nluc were collected after imaging on an Ami HT IVIS on 2 and 4 dpi. After homogenization, Nluc activity (**A**) and viral titers (**B**) in tissue homogenates were determined on a microplate reader or by plaque assay on Vero cells, respectively. The results are the mean values ± SD and analyzed by an unpaired *t*-test. *** *p* < 0.001; **** *p* < 0.0001. LOD, limit of detection.

Quantification of Nluc activity in the lung and nasal turbinate showed a 3-log reduction in Nluc activity at both 2 and 4 dpi in vaccinated mice compared to unvaccinated mice (Figure 6A). Plaque assays confirmed these findings, with lung virus titers in vaccinated mice falling below the limit of detection (LOD) at 4 dpi (Figure 6B). Notably, Nluc activity in the nasal turbinate was undetectable at 4 dpi, highlighting the vaccine’s ability to block viral replication at the primary infection site.

## 4. Discussion

I.N. immunization with the CoPoP-RBD nanoparticle vaccine formulated with KLA and α-GalCer elicited strong mucosal and systemic antibody responses against SARS-CoV-2. Notably, the vaccine induced high levels of RBD-specific IgA in the lungs, whereas I.M. administration did not generate detectable mucosal IgA. This observation highlights the advantage of the I.N. route in promoting local immune responses.

Among the 22 adjuvants screened, the combination of KLA, a TLR4 agonist, and α-GalCer, an iNKT cell agonist, proved to be the most effective, producing the highest antigen-specific IgA and IgG titers. However, the immunological mechanisms by which KLA and α-GalCer synergize to enhance mucosal immunity were not investigated here. The success of this dual-adjuvant strategy reflects a broader principle in mucosal vaccine design: combining immunostimulatory components that target different innate immune pathways can amplify immune responses. Supporting this concept, a recent study utilizing a two-component nasal adjuvant (nanoemulsion combined with an RNA-based RIG-I agonist) also demonstrated robust humoral, mucosal, and cellular immunity in mice [39].

We further optimized the vaccine formulation by evaluating the effects of adjuvant dosage and administration volume. Increasing the I.N. administration volume to 50 µL per mouse significantly enhanced both lung IgA and serum IgG responses compared to a smaller 10 µL volume, likely due to improved antigen dispersion across the nasal mucosa. However, it is worth noting that administration volumes of 20–30 µL in mice tend to deliver vaccines to both the upper and lower respiratory tracts [40], which may not accurately model pure nasal immunization in humans. Therefore, volume considerations represent an important practical factor; while larger volumes improve responses in mice, careful optimization will be required for translation to human vaccination.

The study design carries several limitations: The I.N. vaccine was tested using only the original Wuhan strain of SARS-CoV-2, which may not fully reflect efficacy against currently circulating variants. In this study, we only used mouse models to evaluate vaccine efficacy. Cytokine responses to vaccination can vary substantially across species and immunological contexts. Even in transgenic mice expressing hACE2, the complexity of human immune responses and nasal anatomy is not fully replicated. The adjuvant-induced cytokine profile observed in mice may not directly predict the response in humans. The I.N. use of adjuvants raises safety concerns. Some studies have suggested that α-GalCer can potentially induce liver damage in mice [41] or vaccine-associated enhanced respiratory disease in pigs [42]. A safety study was not carried out, but a careful evaluation of tolerability is needed in the future.

A further limitation of this study is that, for simplicity, all adjuvants were dosed at fixed mass ratios while screening, and their effective dose and therapeutic index were not considered. Therefore, many adjuvants were likely not assessed at their full immunostimulatory potential. In addition, the 50 μL and 10 μL administration volumes did not elicit comparable immune responses, even though we consider 10 μL to be more representative of I.N. immunization since 50 μL likely results in respiratory drainage to the lungs. Further efforts are required to systematically evaluate how administration volume influences vaccine distribution and immunogenicity in intranasal vaccination. Despite these limitations, the dual-adjuvant intranasal strategy substantially increased mucosal IgA levels, although the overall magnitude of the response indicates that there remains room for further enhancement.

Future studies could include tracking of cytokine production and lung immune cell interactions in response to these adjuvants. Such analyses would provide deeper insight into the immune response to KLA and α-GalCer, guiding the development of optimized vaccines.

## 5. Conclusions

In this study, we developed a potent I.N. SARS-CoV-2 subunit vaccine formulation that induces both local and systemic immunity and confers protection against viral challenge. The incorporation of Kdo2-Lipid A and α-GalCer adjuvants into a CoPoP liposome-displayed SARS-CoV-2 RBD nanoparticle was key to achieving high immunogenicity via the I.N. route. Mice immunized with the optimized formulation developed high titers of neutralizing IgG in the serum as well as antigen-specific IgA in the respiratory tract, immune features that are critical for blocking infection at the point of entry. In addition, the incorporation of DOTAP improved mucoadhesion and antigen retention in the respiratory tract. When tested in a susceptible mouse model, the I.N. vaccine effectively limited viral replication in the nasopharynx and lungs, preventing illness and death. These results underscore the significance of adjuvant-enabled mucosal vaccination as a strategy to enhance protection against respiratory pathogens. The I.N. RBD vaccine described here offers a promising approach to supplement existing systemic vaccines by providing an added layer of mucosal immunity, which could reduce transmission and better control COVID-19. Overall, our work highlights the feasibility and benefits of I.N. adjuvant optimization, and it lays the groundwork for further development of needle-free vaccines that safeguard both individual and public health.

## Data Availability

The raw data supporting the conclusions of this article will be made available by the authors on request.

## Supplementary Materials

The following supporting information can be downloaded at: https://www.mdpi.com/article/10.3390/pharmaceutics17111422/s1, Table S1: List of Screened Adjuvants; Table S2: Formulation of vaccines for different charged lipids study; Figure S1: Anti-RBD IgG titer in adjuvants screening; Figure S2: Replication and spread of rSARS-CoV-2-WA1-Nluc in vaccinated K18 hACE2 transgenic mice.; Figure S3: Replication and spread of rSARS-CoV-2 WA1-Nluc in vaccinated K18 hACE2 transgenic mice. Figure S4: Evaluation of I.N. based vaccine candidates’ immunogenicity in K18 hACE2 transgenic mice.
