# Supplementary material for: In Vivo Iterative Adjuvant Screening Identifies an Intranasal Vaccine Formulation for Elicitation of Protective Mucosal Immune Responses Against SARS-CoV-2
Source: Pharmaceutics. 2025 Nov 3;17(11):1422. doi: 10.3390/pharmaceutics17111422 (PMC12655344; doi:10.3390/pharmaceutics17111422)
Supplement: Supplementary file 1 [file pharmaceutics-17-01422-s001.zip › pharmaceutics-3906826-supplementary.pdf]

Supporting Information: In Vivo Iterative Adjuvant Screening Identifies an Intranasal Vaccine Formulation for Elicitation of Protective Mucosal Immune Responses Against SARS-CoV-2

Table S1. List of Screened Adjuvants

Compound	Class	Vendor*	Putative lipid particle binding	Solubility strategy
QS7	Inflammasome	Desert King	Cholesterol binding	Aqueous
QS18	Inflammasome	Desert King	Cholesterol binding	Aqueous
QS21	Inflammasome	Desert King	Cholesterol binding	Aqueous
α-GalCer	iNKT ligand	Avanti (867000)	Lipid-like	Ethanol
DDAB	Cationic & alkyl tail	Avanti (890810)	Lipid-like	Ethanol
Diprovocim (div)	TLR1/2	MedChemExpress (HY-123942)	Hydrophobic	Aqueous
PAM3CSK4	TLR1/2	Invivogen (vac-pms)	Lipid-like	Ethanol
PAM2C-CL401	TLR2/7	Invivogen (vac-c401-5)	Lipid-like	Ethanol
PAM2C-CL413	TLR2/7	Invivogen (vac-c413-5)	Lipid-like	Ethanol
PAM2C-CL429	TLR2/NOD2	Invivogen (vac-c429)	Lipid-like	Ethanol
3D6A-PHAD	TLR4	Avanti (699855)	Lipid-like	Ethanol
PHAD-504	TLR4	Avanti (699810)	Lipid-like	Ethanol
3M052	TLR7/8	MedChemExpress (HY-109104)	Hydrophobic	Ethanol
3M-002(CL075)	TLR8	MedChemExpress (HY-117066)	Hydrophobic	Ethanol
Selgantolimod	TLR8	MedChemExpress (HY-109137)	Hydrophobic	Ethanol
Motolimod	TLR8	MedChemExpress (HY-13773)	Hydrophobic	Ethanol
STING-Agonist-1	STING	MedChemExpress (HY-19711)	Hydrophobic	Ethanol
DSR-6434	TLR7	MedChemExpress (HY-110120)	Hydrophobic	Ethanol
Resiquimod (R848)	TLR7/8	MedChemExpress (HY-13740)	Hydrophobic	Ethanol
Kdo2-lipidA	TLR4	Avanti (699500)	Lipid-like	Ethanol
MPLA	TLR4	Avanti (699800)	Lipid-like	Ethanol
7DW8-5	iNKT ligand		Lipid-like	Ethanol

* Catalog numbers are shown in parentheses where applicable

Table S2: Formulation of vaccines for different charged lipids study

Group	1	2	3	4	5 (IM)
DOPC (μg)	160	80	80	80	160
DOTAP (μg)		80	80		
DOPG (μg)				80	
Chol (μg)	40	40	40	40	40
CoPoP (μg)	8	8	8	8	8
KLA (μg)	2	2		2	
α-GalCer (μg)	2	2		2	
PHAD (μg)					3.2

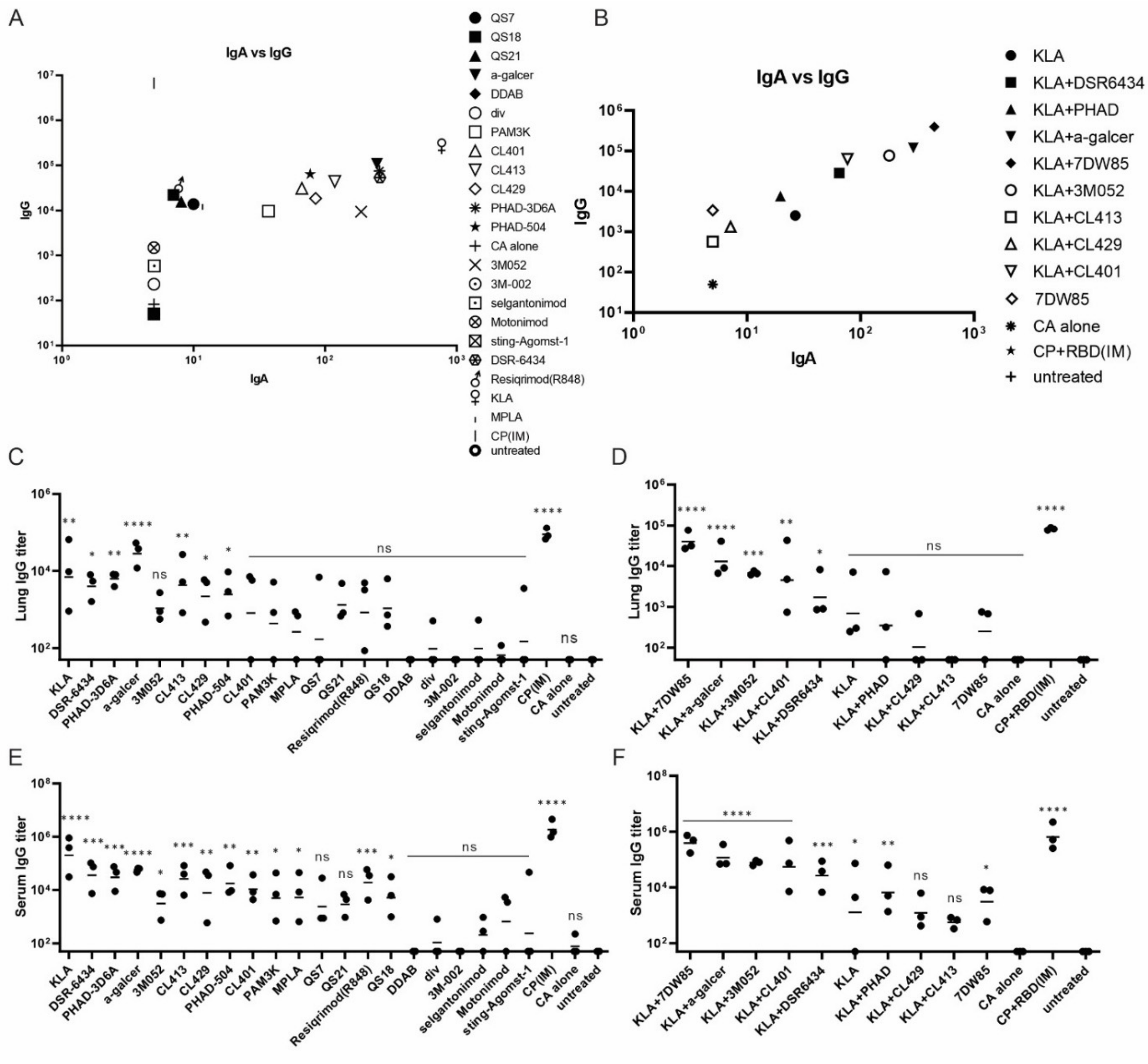

Figure S1: Anti-RBD IgG titer in adjuvants screening. (A) RBD-specific IgA and IgG titers were quantified by ELISA. Each symbol represents a different adjuvant in the first screening as indicated in the legend. The graph displays the mean titers of IgA (x-axis) and IgG (y-axis) for each group. **(B)** The mean titers of IgA and IgG in the second round of screening. **(C)** The IgG titer measured by mice lung homogenates in the first screening and **(D)** the second screening. **(E)** The IgG titer measured by mice serum in the first screening and **(F)** the second screening. Log10-transformed IgG titer (C, D, E, F) were

analyzed based on one-way ANOVA followed by Dunnett multiple comparison test. *p < 0.05; **p < 0.01; ***p < 0.001; ****p < 0.0001; ns: no statistical.

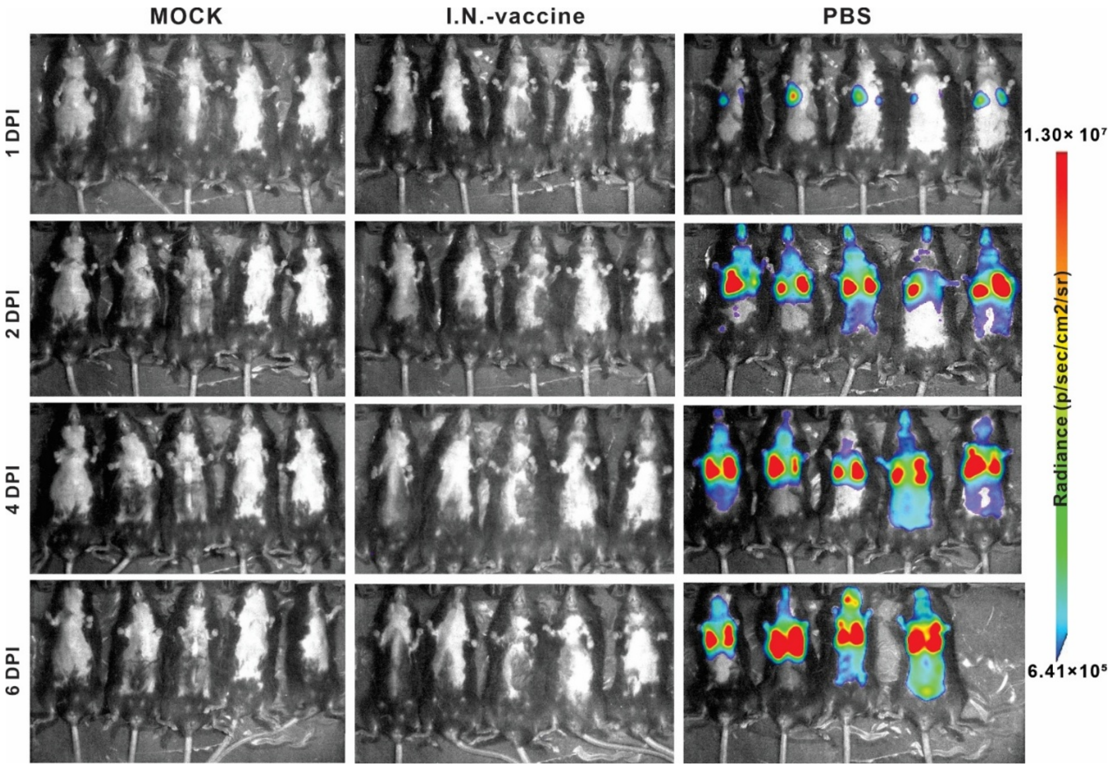

Figure S2: Replication and spread of rSARS-CoV-2-WA1-Nluc in vaccinated K18 hACE2 transgenic mice. 6-8-week-old K18 hACE2 transgenic mice (n=5/group) were mock-infected or infected I.N. with 10^5 PFU/mouse of rSARS-CoV-2-WA1-Nluc. Nluc activity in the whole mouse at the indicated dpi was evaluated with an Ami HT *in vivo* imaging system (IVIS). Representative images of the same mouse at 1, 2, 4, and 6 dpi are shown.

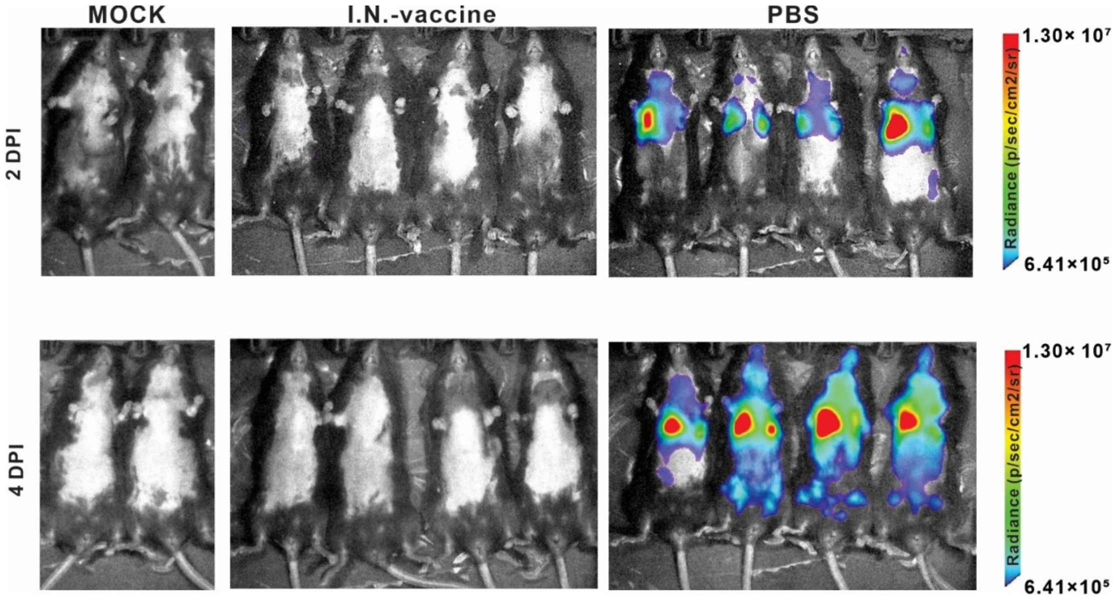

Figure S3: Replication and spread of rSARS-CoV-2 WA1-Nluc in vaccinated K18 hACE2 transgenic mice. *In vivo* Nluc activity in live mice (n=4/group) mock-infected or infected (10^5 PFU/mouse) with rSARS-CoV-2-WA1-Nluc were determined on 2 and 4 dpi using the Ami HT IVIS.

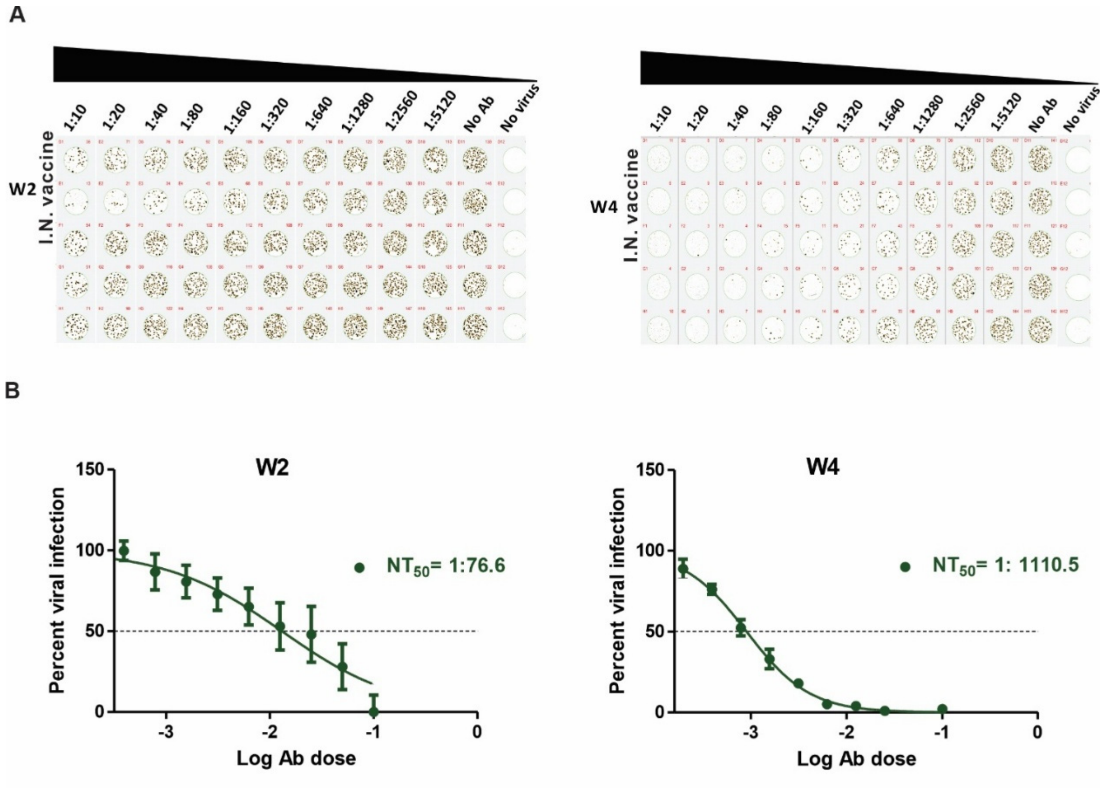

Figure S4: Evaluation of I.N. based vaccine candidates' immunogenicity in K18 hACE2 transgenic mice: detection of SARS-CoV-2 neutralizing antibodies in sera at 14 days post-prime (d.p.p.) and 14 days post-boost (d.p.b). PRMNT assay to identify SARS-CoV-2 neutralizing antibodies (NAbs). **(A)** 2-fold serially diluted serum samples are pre-incubated with ~100-200 PFU of rSARS-CoV-2-WA1-Nluc for 1 h at 37 °C. After 1 h pre-incubation, confluent monolayers of Vero AT cells (96-well plate format, ~4 x 10^4 cells/well) were infected with the serum-virus mixture for 1 h. Cells in row 11 were incubated with virus only and cells in row 12 are mock-infected and used as internal controls in each plate. After 1 h of virus adsorption, post-infection media containing 1% Avicel was added to all the wells. At 14 hours post-infection (h.p.i.), cells were fixed with 10% formalin solution. After 24 h fixation, cells

were washed 3X with water and incubated with 1 μg/mL of a SARS-CoV-1 cross-reactive nucleocapsid (N) protein mAb (1C7C7) at 37 °C. After 1 h incubation with the primary mAb, cells were washed 3X with water and incubated with a secondary POD anti-mouse Ab (diluted according to the manufacturer's instruction) at 37 °C. After 30 minutes of incubation with the secondary Ab, cells were washed 3X with water and developed with the DAB substrate kit. Positive staining plaques in each of the wells were quantified using an ELISPOT plate reader. **(B)** The NT_{50} was calculated as the highest dilution of the sera that prevents 50% plaque formation in infected cells. The dotted line indicates 50% neutralization (NT_{50}).
